# Antibacterial efficacy of indigenous Pakistani honey against extensively drug-resistant clinical isolates of *Salmonella enterica* serovar Typhi: an alternative option to combat antimicrobial resistance

**DOI:** 10.1186/s12906-023-03870-8

**Published:** 2023-02-08

**Authors:** Hasan Ejaz, Mamoona Sultan, Muhammad Usman Qamar, Kashaf Junaid, Nasir Rasool, Awadh Alanazi, Mashael W. Alruways, Bi Bi Zainab Mazhari, Yasir Alruwaili, Syed Nasir Abbas Bukhari, Sonia Younas

**Affiliations:** 1 Department of Clinical Laboratory Sciences, College of Applied Medical Sciences, Jouf University, Sakaka, 72388 Saudi Arabia; 2 Institute of Microbiology, Faculty of Life Sciences, Government College University Faisalabad, Faisalabad, 38000 Pakistan; 3 School of Biological and Behavioural Sciences, Queen Mary University of London, London, E1 4NS UK; 4 Department of Chemistry, Government College University Faisalabad, Faisalabad, 38000 Pakistan; 5 Department of Clinical Laboratory Sciences, College of Applied Medical Sciences, Shaqra University, Shaqra, 15273 Saudi Arabia; 6 Department of Clinical Laboratory Sciences, College of Applied Medical Sciences, Jouf University, Qurayyat, 75911 Saudi Arabia; 7 Department of Pharmaceutical Chemistry, College of Pharmacy, Jouf University, Sakaka, 72388 Al Jouf Saudi Arabia; 8 School of Public Health, LKS Faculty of Medicine, HKU-Pasteur Research Pole, The University of Hong Kong, Hong Kong, China

**Keywords:** Antimicrobial resistance, Natural antibiotics, XDR *S*. Typhi, MIC, Honey, Resistance genes

## Abstract

**Background:**

Extensively drug-resistant (XDR) *Salmonella enterica* serovar Typhi (*S.* Typhi) poses a grave threat to public health due to increased mortality and morbidity caused by typhoid fever. Honey is a promising antibacterial agent, and we aimed to determine the antibacterial activity of honey against XDR *S.* Typhi.

**Methods:**

We isolated 20 clinical isolates of XDR *S*. Typhi from pediatric septicemic patients and determined the minimum inhibitory concentrations (MICs) of different antibiotics against the pathogens using the VITEK 2 Compact system. Antimicrobial-resistant genes carried by the isolates were identified using PCR. The antibacterial efficacy of five Pakistani honeys was examined using agar well diffusion assay, and their MICs and minimum bactericidal concentrations (MBCs) were determined with the broth microdilution method.

**Results:**

All 20 isolates were confirmed as *S.* Typhi. The antibiogram phenotype was confirmed as XDR *S.* Typhi with resistance to ampicillin (≥ 32 µg/mL), ciprofloxacin (≥ 4 µg/mL), and ceftriaxone (≥ 4 µg/mL) and sensitivity to azithromycin (≤ 16 µg/mL) and carbapenems (≤ 1 µg/mL). Molecular conformation revealed the presence of *bla*_TM-1_*, Sul1, qnrS, gyrA, gyrB,* and *bla*_CTX-M-15_ genes in all isolates. Among the five honeys, beri honey had the highest zone of inhibition of 7–15 mm and neem honey had a zone of inhibition of 7–12 mm. The MIC and MBC of beri honey against 3/20 (15%) XDR *S*. Typhi isolates were 3.125 and 6.25%, respectively, while the MIC and MBC of neem were 3.125 and 6.25%, respectively, against 3/20 (15%) isolates and 6.25 and 12.5%, respectively, against 7/20 (35%) isolates.

**Conclusion:**

Indigenous honeys have an effective role in combating XDR *S*. Typhi. They are potential candidates for clinical trials as alternative therapeutic options against XDR *S*. Typhi isolates.

## Background

Typhoid fever is a fatal disease caused by *Salmonella enterica* serovar Typhi (*S*. Typhi), which is usually transmitted via contaminated food and water. It results in an extended hospital stay, an additional financial burden, and high mortality among vulnerable individuals [1]. The World Health Organization (WHO) has estimated that 11 to 20 million people are infected with *S*. Typhi, among whom, 128,000 to 161,000 die every year [2]. The heaviest burden of typhoid fever is reported in South Asia and Africa due to the unavailability of clean drinking water and fragile health systems [3]. The rate of incidence of typhoid fever in Pakistan stands at 493.5/100,000 individuals per year [4].

The first case of ceftriaxone resistance in *S*. Typhi was reported in Bangladesh in 1999 [5]. The first epidemic of extensively drug-resistant (XDR) *S*. Typhi was reported in Hyderabad, Sindh Province, Pakistan, in 2016 [6]. Several *S*. Typhi pathogens are resistant to antibiotics normally recommended for the treatment of typhoid fever, including ampicillin, ceftriaxone, chloramphenicol, ciprofloxacin, and sulfamethoxazole/trimethoprim, and are only sensitive to azithromycin (oral antibiotic) and carbapenems (injectable antibiotic) [7]. However, even cases of azithromycin resistance have been reported from Bangladesh [8], Nepal [9], and India [10]. Multidrug-resistant (MDR) *S*. Typhi pathogens frequently carry the plasmid-mediated *bla*_TEM-1_*, dhfR7, sul1,* and *catA1* genes and extensively drug-resistant (XDR) *S*. Typhi carry *parE* and *bla*_CTX-M-15_ antimicrobial resistance genes (ARGs) [11]. A recent Pakistani study revealed the spread of MDR (50.1%) and XDR (33%) *S*. Typhi in pediatric septicemic patients [12]. These XDR strains have also been reported in other parts of the world with a travel history to Pakistan, including the United States [13], the United Kingdom [14], Australia [15], Denmark [16], and Canada [17].

The WHO has classified *S.* Typhi as a high-priority pathogen against which new treatment options are urgently needed [18]. Honey is well recognized for its anti-inflammatory and antibacterial properties against various MDR pathogens. Several factors contribute to the antimicrobial properties of honey, including methylglyoxal, an acidic pH, 40 – 75% sugar contents, high osmotic effects, and the presence of bacteriostatic and bactericidal factors, including antioxidants, hydrogen peroxide, lysozyme, phosphates, polyphenols, flavonoids, phenolic acid, immuno-regulating properties, and trace elements [19, 20]. Unlike traditional antibiotics, honey promotes the growth of bifidobacteria and lactobacilli in the stomach rather than interfering with the growth of beneficial gastric bacteria. Indigenous Pakistani honey has rarely been studied for its antibacterial properties against Gram-negative bacteria, particularly XDR *S.* Typhi [21]. Therefore, in this study, we aimed to determine the antibacterial activity of native honey against the molecularly characterized clinical isolates of XDR *S*. Typhi harboring several drug-resistant genes.

## Materials and methods

### Study design and setting

This study was prospectively conducted at the Department of Microbiology of Government College University, Faisalabad, Pakistan. Clinical isolates of XDR *Salmonella* Typhi were obtained from a tertiary care hospital in Lahore, Pakistan, between October 2021 and February 2022. The study was ethically approved by the Ethical Review Committee (ERC) of the university and conducted in accordance with the World Medical Association’s Declaration of Helsinki.

### Collection of samples

Bacterial isolates were collected from suspected septicemic pediatric patients. Briefly, blood samples (1–3 mL) obtained from children were cultured in pediatric BD BACTEC™ blood culture bottles and placed in the BD BACTEC™ blood culture automated instrument (BD, Pont-de-Claix, France) for up to five days. The instrument detected bacterial growth during incubation, and the positive samples were further cultured for identification of bacteria.

### Isolate confirmation

The bacterial isolates were sub-cultured on *Salmonella Shigella* agar (SSA), and the plates were incubated at 37 °C overnight in an aerobic environment. The isolates were first identified by bacterial morphology, Gram staining, oxidase, and biochemical reactions and confirmed by the compact VITEK 2 system (bioMérieux, Marcy-l'Étoile, France). Twenty clinical isolates of XDR *S*. Typhi obtained from the clinical settings were preserved in 15% glycerol at -80 °C.

### Minimum inhibitory concentrations of antibiotics against *S*. Typhi

The minimum inhibitory concentration (MIC) against *S*. Typhi isolates was determined for ampicillin, trimethoprim/sulfamethoxazole, third-generation cephalosporin (ceftriaxone), chloramphenicol, ciprofloxacin, azithromycin, imipenem, and meropenem using the VITEK 2 Compact system (bioMerieux, Marcy-l'Étoile, France). Furthermore, the MIC of azithromycin was obtained with broth microdilution assay as described by the Clinical and Laboratory Standards Institute guidelines [22].

### Molecular identification of XDR *S*. Typhi genes

Bacterial DNA was extracted with a commercial DNA extraction kit (Qiagen, Manchester, United Kingdom), and ARGs were detected using molecular techniques. The primer sequences used for molecular identification of ARGs are listed in Table 1. We used Phusion High-Fidelity DNA Polymerase (Thermo Fisher Scientific Inc., Waltham, United States) for PCR amplification of the ARGs [11]. The amplification process began with denaturation of DNA at 95 °C for 4 min followed by 30 cycles of denaturation at 95 °C for 30 s, annealing at 55 °C for 35 s, and extension at 72 °C for 7 min on a T100 thermal cycler (Bio-Rad, Hercules, USA). The PCR products were run on 1.5% agarose gel with SYBR Safe (Invitrogen, Waltham, United States) gel stain in conjunction with the positive and negative controls [23]. The gene products were compared to GeneRuler 100 bp DNA Ladder (Thermo Fisher Scientific Inc., Waltham, United States). The amplified ARGs were observed on the transilluminator (Fisher Scientific International, Inc., Hampton, United States), and a PCR cleanup kit was used to clean the amplified products before sequencing [24]. The DNA sequence data were analyzed using the National Center for Biotechnology Information (NCBI) database.

**Table 1:** The primers sequences used for molecular identification of antibiotic resistance genes [11]

Gene	Forward primers (5' - 3')	Reverse primers (5' - 3')
*pltB*	TAAACCATGATAGACTGG	GAAAGTTACGGTTATACC
*bla*_CTX-15_	GATGTGCAGCACCAGTAAAG	AACGATATCGCGGTGATCT
*Sul1*	GTATTGCGCCGCTCTTAGAC	AGGGTTTCCGAGAAGGTGAT
*bla*_TEM-1_	AACCCTGGTAAATGCTTC	GTATATATGAGTAAACTTGG
*qnrS*	TATAATGGTAGTCTAGCCC	GATGTGTGATTTTAAACG
*qnrA*	CTTTGAATCCGGGATACAG	TTCCATAGACAAGAAAAAGG
*qnrB*	GAAAAGGGTAAAATAACGG	CATCATGATGCCCTGGCCAG

### Collection of indigenous honeys

Five native honeys of different botanical origins—beri honey (*Ziziphus mauritiana*), neem honey (*Azadirachta indica*), sidr honey (*Ziziphus spina-christi*), orange honey (*Citrus sinensis*), and mustard honey (*Brassica nigra*)—were collected from different geographic locations in Pakistan and their effects against XDR *S*. Typhi were studied. Samples were collected from commercial bee producers. The identification of the plant source of the honey samples was based on geographic location, flowering plant, flavor, season, and color of each honey. Samples (250 g) of each honey in sterile containers were obtained directly from the beekeeper and placed in the dark at room temperature.

### Agar well diffusion assay of honeys

The antibacterial activity of the natural honeys against XDR *S*. Typhi was assessed using agar well diffusion analysis as described previously [25]. In brief, a bacterial suspension (0.5 McFarland) was prepared and inoculated on to a plate of Mueller Hinton agar (MHA). Six wells were made in MHA with a sterile 6-mm cork borer. Each indigenous honey was serially diluted (v/v%) in distilled water to 50, 40, 30, 20, and 10% in separate sterile test tubes. We added 120 µL of each honey dilution into the respective wells in MHA. The plates were aerobically incubated at 37 ºC for 18–20 h. The zone of inhibition (mm) of each well was measured using vernier calipers. The assay was performed in triplicate.

### Determination of minimum inhibitory concentration of honeys

Broth microdilution assays were performed to determine the MICs (v/v) of the different honeys against XDR *S*. Typhi isolates in 96-well microtitration plates (Thermo Fisher Scientific Inc., Waltham, United States) [25]. We prepared a 0.5 McFarland bacterial suspension by mixing bacterial colonies in 3 mL sterile normal saline. We added 100 µL of sterile nutrient broth to each of wells 1–12 of the microtiter plate, and 100 µL of 0.5 McFarland bacterial suspension was added to each of wells 1–10. The last two wells were used for the positive and negative controls. The negative control well contained only 100 µL of honey. Later, 100 µL of 50% honey was added into the first well and was double diluted in the succeeding wells to 25, 12.5, 6.25, 3.125, 1.56, 0.78, 0.39, 0.195, and 0.0975%. The plates were aerobically incubated overnight in an incubator shaker at 37 °C. MIC (%v/v) was calculated by comparing the positive and negative control wells. The procedures were executed in triplicate.

### Determination of minimum bactericidal concentration of honeys

The minimum bactericidal concentration (MBC) was determined by counting the colony-forming unit on the nutrient agar plates. After determining the MIC, 10 µL of bacterial suspension was taken from each well of the microtitration plate and inoculated on to nutrient agar. The plates were aerobically incubated for 24 h at 37 °C. The colony count of each plate was determined and scored as bacterial growth. All procedures were performed in triplicate [25].

## Results

### Antimicrobial susceptibility of XDR *S*. Typhi

Twenty isolates from pediatric septicemic patients were confirmed as *S*. Typhi. Antimicrobial susceptibility testing showed that the MICs (µg/mL) of ampicillin (≥ 32 µg/mL), co-trimoxazole (≥ 4/76 µg/mL), ciprofloxacin (≥ 4 µg/mL), and ceftriaxone (≥ 4 µg/mL) were higher against all 20 isolates. All isolates were sensitive to oral azithromycin (≤ 16 µg/mL) (Table 2).

**Table 2:** Minimum inhibitory concentration (MIC; µg/mL) of antibiotics against XDR *S*. Typhi isolates

*S*. Typhi isolates	MIC breakpoint of antibiotics						
	**AMP** ≤ 8 to ≥ 32	**SXT** ≤ 2/38 to ≥ 4/76	**CIP** ≤ 1 to ≥ 4	**CRO** ≤ 1 to ≥ 4	**AZM** ≤ 16 to ≥ 32	**IPM** ≤ 1 to ≥ 4	**MEM** ≤ 1 to ≥ 4
ST-1	≥ 32	≥ 4/76	≥ 4	32	8	0.5	0.5
ST-2	≥ 32	≥ 4/76	≥ 4	≥ 64	4	0.25	0.5
ST-3	≥ 32	≥ 4/76	≥ 4	≥ 4	4	0.5	0.25
ST-4	≥ 32	≥ 4/76	≥ 4	16	4	0.5	0.5
ST-5	≥ 32	≥ 4/76	≥ 4	16	2	0.25	0.5
ST-6	≥ 32	≥ 4/76	≥ 4	≥ 4	2	0.5	0.5
ST-7	≥ 32	≥ 4/76	≥ 4	≥ 4	8	0.25	0.5
ST-8	≥ 32	≥ 4/76	≥ 4	≥ 4	2	0.5	0.5
ST-9	≥ 32	≥ 4/76	≥ 4	≥ 64	2	0.25	0.25
ST-10	≥ 32	≥ 4/76	≥ 4	16	2	0.5	0.5
ST-11	≥ 32	≥ 4/76	≥ 4	8	4	0.25	0.5
ST-12	≥ 32	≥ 4/76	≥ 4	8	4	0.5	0.5
ST-13	≥ 32	≥ 4/76	≥ 4	8	2	0.25	0.5
ST-14	≥ 32	≥ 4/76	≥ 4	≥ 64	8	25	25
ST-15	≥ 32	≥ 4/76	≥ 4	≥ 4	2	0.5	0.5
ST-16	≥ 32	≥ 4/76	≥ 4	16	4	0.5	0.25
ST-17	≥ 32	≥ 4/76	≥ 4	32	8	0.5	0.5
ST-18	≥ 32	≥ 4/76	≥ 4	≥ 4	8	0.5	0.5
ST-19	≥ 32	≥ 4/76	≥ 4	≥ 4	4	0.25	0.5
ST-20	≥ 32	≥ 4/76	≥ 4	≥ 4	8	0.5	0.5

*AMP* Ampicillin, *SXT* Co-trimoxazole, *CIP* Ciprofloxacin, *CRO* Ceftriaxone, *AZM* Azithromycin, *IPM* Imipenem, *MEM* Meropenem

### Molecular confirmation of XDR *S*. Typhi

Molecular confirmation using PCR showed that the isolates were simultaneously positive for multiple ARGs. All MDR and XDR *S*. Typhi isolates harbored the resistance genes *pltB, bla*_CTX-M-15_*, bla*_TEM-1_*, qnrS, qnrA, qnrB,* and *Sul1* (Table 3).

**Table 3:** Molecular confirmation of resistance genes in XDR *S*. Typhi

Isolates	*bla*_TEM-1_	*Sul1*	*qnrA*	*qnrB*	*qnrS*	*pltB*	*bla*_CTX-M-15_
ST-1	** + **	** + **	** + **	** + **	** + **	** + **	** + **
ST-2	** + **	** + **	** + **	** + **	** + **	** + **	** + **
ST-3	** + **	** + **	** + **	** + **	** + **	** + **	** + **
ST-4	** + **	** + **	** + **	** + **	** + **	** + **	** + **
ST-5	** + **	** + **	** + **	** + **	** + **	** + **	** + **
ST-6	** + **	** + **	** + **	** + **	** + **	** + **	** + **
ST-7	** + **	** + **	** + **	** + **	** + **	** + **	** + **
ST-8	** + **	** + **	** + **	** + **	** + **	** + **	** + **
ST-9	** + **	** + **	** + **	** + **	** + **	** + **	** + **
ST-10	** + **	** + **	** + **	** + **	** + **	** + **	** + **
ST-11	** + **	** + **	** + **	** + **	** + **	** + **	** + **
ST-12	** + **	** + **	** + **	** + **	** + **	** + **	** + **
ST-13	** + **	** + **	** + **	** + **	** + **	** + **	** + **
ST-14	** + **	** + **	** + **	** + **	** + **	** + **	** + **
ST-15	** + **	** + **	** + **	** + **	** + **	** + **	** + **
ST-16	** + **	** + **	** + **	** + **	** + **	** + **	** + **
ST-17	** + **	** + **	** + **	** + **	** + **	** + **	** + **
ST-18	** + **	** + **	** + **	** + **	** + **	** + **	** + **
ST-19	** + **	** + **	** + **	** + **	** + **	** + **	** + **
ST-20	** + **	** + **	** + **	** + **	** + **	** + **	** + **

### Agar well diffusion assay for XDR *S*. Typhi

**Fig. 1:**
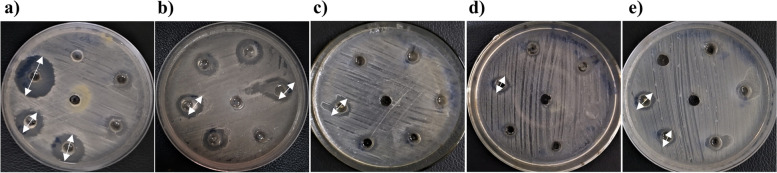
The zones of inhibition (mm) of honey against XDR *S. *Typhi using the agar well diffusion assay. The figure illustrates the variable zones of inhibition of (**a**) beri, (**b**) neem, (**c**) sidr, (**d**) orange and (**e**) mustard honey. The arrows indicate examples of inhibition zones

The agar well diffusion assay showed different zones of inhibition for the five honeys (Fig. 1). The maximum inhibition zones against XDR *S*. Typhi isolates were observed for beri honey. Beri honey displayed an inhibition zone of 7–15 mm. One isolate showed an inhibition zone of 15 mm, followed by two isolates with 14 mm, one with 13 mm, and three with 12 mm. The zone of inhibition for neem honey ranged from 7 to 12 mm, with two isolates showing a zone of inhibition of 12 mm, two of 11 mm, and five of 10 mm. Furthermore, sidr honey had an inhibition zone ranging from 7 to 12 mm, with three isolates exhibiting an inhibition zone of 12 mm, two of 11 mm, and two of 10 mm. However, orange honey and mustard honey showed comparatively small inhibition zones (Table 4).

**Table 4:** Antibacterial activity (zone of inhibition; mm) of five indigenous honeys against XDR *S*. Typhi

XDR *S.* Typhi isolates	Beri honey	Neem honey	Sidr honey	Orange honey	Mustard honey
ST-1	10 ± 2.0	11 ± 2.3	8 ± 2.0	6 ± 2.0	5 ± 1.9
ST-2	14 ± 2.7	10 ± 2.3	8 ± 1.9	10 ± 2.7	6 ± 2.4
ST-3	15 ± 2.0	9 ± 1.3	8 ± 1.9	6 ± 2.4	6 ± 2.4
ST-4	9 ± 1.3	12 ± 2.1	7 ± 1.7	5 ± 1.9	5 ± 1.9
ST-5	14 ± 2.0	10 ± 2.2	12 ± 1.0	9 ± 2.5	6 ± 2.2
ST-6	8 ± 1.6	7 ± 1.9	8 ± 1.7	7 ± 2.4	5 ± 2.0
ST-7	12 ± 1.2	10 ± 2.1	12 ± 2.2	7 ± 2.3	5 ± 2.0
ST-8	10 ± 1.7	6 ± 2.3	9 ± 2.2	7 ± 2.0	5 ± 2.0
ST-9	7 ± 2.1	10 ± 2.4	10 ± 1.9	7 ± 1.7	7 ± 1.7
ST-10	8 ± 2.1	7 ± 2.2	8 ± 2.0	8 ± 2.1	7 ± 1.9
ST-11	13 ± 2.0	11 ± 2.3	11 ± 2.0	8 ± 2.0	5 ± 1.8
ST-12	12 ± 2.7	9 ± 2.3	9 ± 1.9	8 ± 2.7	5 ± 1.8
ST-13	10 ± 2.0	9 ± 2.2	7 ± 1.9	8 ± 2.4	7 ± 2.0
ST-14	9 ± 1.3	12 ± 2.1	7 ± 1.7	9 ± 1.9	6 ± 1.9
ST-15	9 ± 1.3	8 ± 2.2	11 ± 1.0	8 ± 2.5	5 ± 1.5
ST-16	10 ± 1.6	8 ± 1.9	7 ± 1.7	8 ± 2.4	6 ± 1.9
ST-17	12 ± 1.2	10 ± 2.1	12 ± 2.2	9 ± 2.3	5 ± 2.5
ST-18	10 ± 1.7	7 ± 2.3	4 ± 2.2	8 ± 2.0	6 ± 1.8
ST-19	7 ± 2.1	7 ± 2.4	10 ± 1.9	6 ± 2.4	5 ± 1.7
ST-20	11 ± 2.1	8 ± 2.2	3 ± 2.0	7 ± 2.1	6 ± 2.0

### MICs of indigenous honeys against XDR *S*. Typhi isolates

**Fig. 2:**
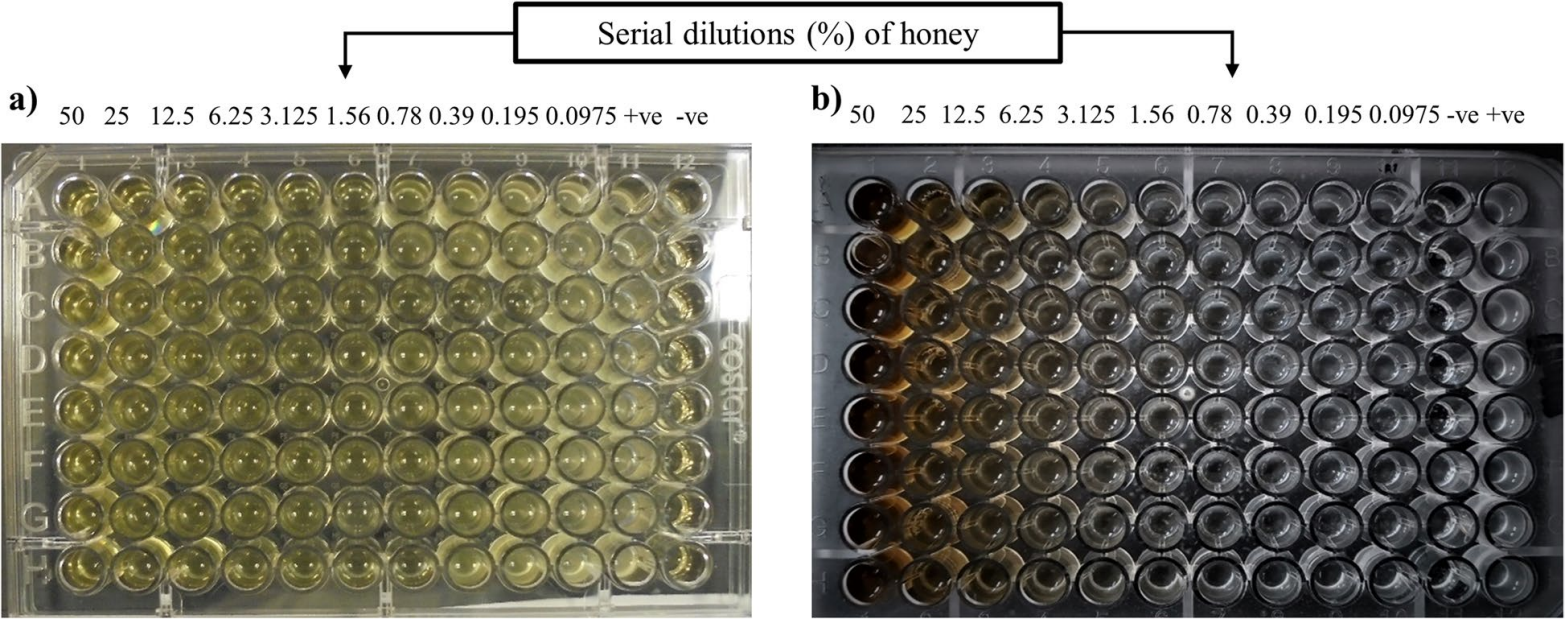
The microtitration plates show different concentrations of honey used against XDR *S*. Typhi isolates. **a** The plate shows the minimum inhibitory concentration (MIC) of beri honey at different serial dilutions against XDR *S*. Typhi in wells 1 to 10, positive control in well 11, and negative control in well 12. **b** MIC of neem honey at different serial dilutions against XDR *S*. Typhi from wells 1 to 10, negative control in well 11, and positive control in well 12

The broth microdilution assay was used to determine the MICs (v/v%) of native honeys. We found that 3/20 (15%) of the XDR *S*. Typhi isolates were inhibited by beri honey at a low concentration of 3.125%, followed by 9/20 (45%) at 6.25% and 7/20 (35%) at 12.5%. Neem honey inhibited 3/20 (15%) isolates at a concentration of 3.125%, 7/20 (35%) at 6.25%, and 4/20 (20%) at 12.5%. Furthermore, sidr honey inhibited 2/20 (10%) of the isolates at a concentration of 3.125%, followed by 10/20 (50%) at 6.25% and 4/20 (20%) at 12.5%. Orange honey also inhibited 2/20 (10%) XDR isolates at a concentration of 3.125%, followed by 10/20 (50%) at 6.25% and 6/20 (30%) at 12.5%. Mustard honey inhibited 4/20 (20%) of XDR isolates at a concentration of 6.25%, followed by 6/20 (30%) at 6.25% and 6/20 (30%) at 12.5% (Table 5 and Fig. 2).

**Table 5:** Minimum inhibitory concentrations (MICs) and minimum bactericidal concentrations (MBCs) (v/v%) of indigenous honeys against XDR *S.* Typhi isolates

XDR *S.* Typhi isolates	Beri honey		Neem honey		Sidr honey		Orange honey		Mustard honey	
	**MIC**	**MBC**	**MIC**	**MBC**	**MIC**	**MBC**	**MIC**	**MBC**	**MIC**	**MBC**
ST-1	12.5	25	25	50	6.25	12.5	12.5	25	25	50
ST-2	12.5	25	25	50	25	50	6.25	12.5	12.5	25
ST-3	6.25	12.5	12.5	25	6.25	12.5	6.25	12.5	25	50
ST-4	12.5	25	6.25	12.5	6.25	12.5	3.125	6.25	6.25	12.5
ST-5	3.125	6.25	6.25	12.5	12.5	25	6.25	12.5	6.25	12.5
ST-6	6.25	12.5	25	50	6.25	12.5	6.25	12.5	25	50
ST-7	3.125	6.25	25	50	3.125	6.25	12.5	25	6.25	12.5
ST-8	6.25	12.5	6.25	12.5	6.25	12.5	12.5	25	12.5	25
ST-9	6.25	12.5	3.125	6.25	6.25	12.5	6.25	12.5	25	50
ST-10	12.5	25	12.5	25	25	50	6.25	12.5	12.5	25
ST-11	12.5	25	25	50	25	50	6.25	12.5	25	50
ST-12	12.5	25	12.5	25	25	50	12.5	25	25	50
ST-13	25	50	12.5	25	3.125	6.25	6.25	12.5	25	50
ST-14	6.25	12.5	6.25	12.5	12.5	25	25	50	12.5	25
ST-15	6.25	12.5	6.25	12.5	6.25	12.5	25	50	25	50
ST-16	3.125	6.25	6.25	12.5	6.25	12.5	6.25	12.5	12.5	25
ST-17	6.25	12.5	25	50	12.5	25	12.5	25	6.25	12.5
ST-18	12.5	25	6.25	12.5	6.25	12.5	3.125	6.25	25	50
ST-19	6.25	12.5	3.125	6.25	6.25	12.5	12.5	25	12.5	25
ST-20	6.25	12.5	3.125	6.25	12.5	25	6.25	12.5	25	50

### MBC of indigenous honey against XDR *S*. Typhi

**Fig. 3:**
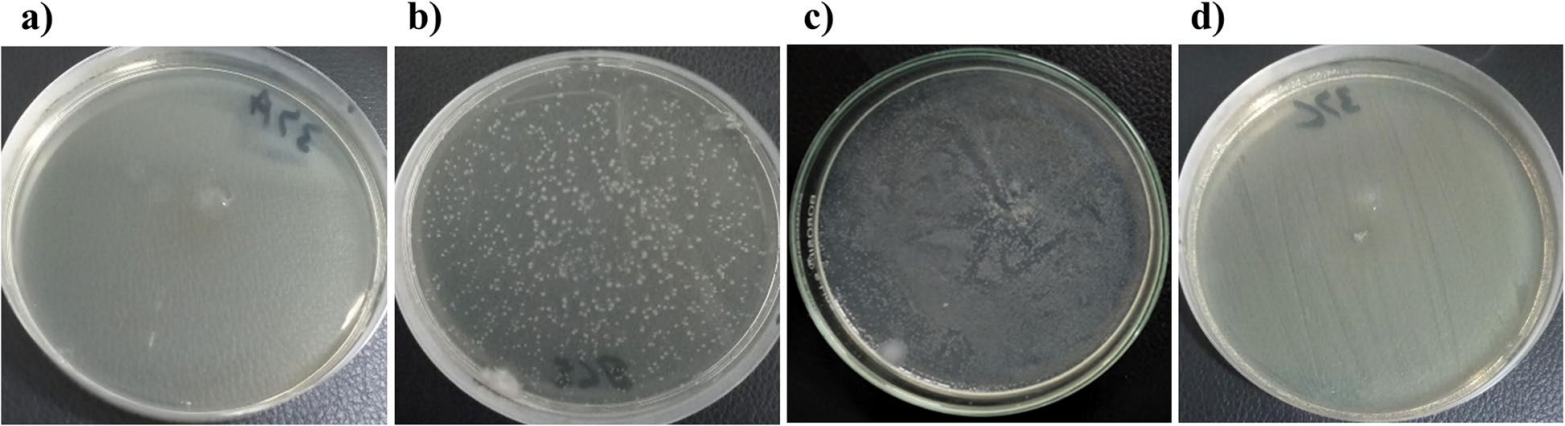
Determination of minimum bactericidal concentration using agar plating at different concentrations (**a**) 50%; (**b**) 25%; (**c**) 12.5; (**d**) 6.25% of honey

The MBC is the first growth-free dilution on an agar plate. Beri honey killed 3/20 (15%) XDR *S.* Typhi isolates at a low concentration of 6.25%, 9/20 (45%) isolates at 12.5%, and 7/20 (35%) isolates at 25%. Neem honey killed 3/20 (15%) isolates at a concentration of 6.25%, 7/20 (35%) isolates at 12.5%, and 4/20 (20%) isolates at 25%. Sidr honey killed 2/20 (10%) isolates at a concentration of 6.25%, 10/20 (50%) isolates at 12.5%, and 4/20 (20%) isolates at 25%. Orange honey also killed 2/20 (10%) XDR *S.* Typhi isolates at a concentration of 6.25%, 10/20 (50%) isolates at 12.5%, and 6/20 (30%) isolates at 25%. Mustard honey killed 4/20 (20%) XDR *S.* Typhi isolates at a concentration of 12.5%, 6/20 (30%) isolates at 12.5%, and 6/20 (30%) isolates at 25% (Table 5 and Fig. 3).

## Discussion

The emergence of XDR *S*. Typhi is a serious global public health issue, particularly in developing countries in Africa and Asia. These pathogens only cause human infections and are also related to systemic infections, especially in vulnerable individuals [26]. In this study, all isolates of *S*. Typhi were identified from blood culture samples obtained from children under 5 years of age. These patients had sepsis with high-grade fever and high pulse and heart rates. Nearly similar results were obtained in an earlier study on sepsis in children in Lahore, Pakistan, which reported an *S*. Typhi prevalence of 10% among children suspected of septicemia [12].

Antimicrobial resistance is a serious global problem, and 10 million people may die by 2050 if it is left unaddressed [27]. *S*. Typhi has progressively become MDR and XDR to several classes of antibiotics, including to antibiotics in the first, second, and third generations [11, 28]. All *S*. Typhi isolates in this study were confirmed as XDR *S*. Typhi with resistance to ampicillin, quinolones, fluoroquinolones, and third-generation cephalosporin and sensitivity to only azithromycin and carbapenems. Likewise, in the first identified case of XDR *S*. Typhi, the isolate was resistant to third-generation cephalosporin and sensitive to only azithromycin and carbapenem [6]. Several studies have documented the spread of MDR and XDR *S*. Typhi pathogens in Pakistan and other parts of the world with a travel history to Pakistan [7, 13–15, 29]. MDR and XDR *S*. Typhi are resistant to several antibiotic classes due to the acquisition of ARGs, including *sul1, qnrS, gyrA, gyrB,* and *bla*_CTX-15_*.* It is widely known that the plasmid-mediated *bla*_CTXM-15_ gene, which has been transmitted to *S*. Typhi from other Enterobacterales, makes *S*. Typhi XDR [30]. The presence of MDR and XDR pathogens is primarily due to the overuse of antibiotics. Azithromycin is one of the drugs excessively used during the COVID-19 pandemic globally [31–33], and the emergence of *S*. Typhi resistant to azithromycin has been reported in different parts of the world [9, 34].

The emergence of antimicrobial resistance throughout the world, in combination with the increasing unavailability of active antimicrobial agents against MDR isolates, necessitates the development of alternative antibiotic strategies. Several molecules and natural extracts have been studied for their potential as new therapeutic weapons in the fight against antibiotic resistance. The essential oil of propolis diminishes the biomass of the biofilm and destroys its structural integrity, thereby impairing cell viability [35]. It has been demonstrated in laboratory experiments that Juniperus extracts have antibacterial and antifungal properties [36]. It has been found that essential oils significantly inhibit the formation of biofilms on food surfaces [37]. Many natural products with antimicrobial properties, particularly honey, are being studied for potential topical application due to the need for more effective therapeutic approaches [38]. Numerous studies conducted on honey, including manuka honey, have established its antibacterial nature against Gram-positive and Gram-negative resistant pathogens [25, 39, 40]. Interestingly, microorganisms are incapable of developing resistance to honey, in contrast to the widespread resistance to synthetic antibiotics. The chemical composition of honey is an important attribute that may account for its antimicrobial nature. In general, all honeys demonstrate antioxidant activity against pathogens as well as polyphenols, flavonoids, and vitamin C [19, 41]. However, research from Turkey, the United States, and Iran indicates that the stability of honey compounds, such as antioxidant capacity and phenolics, may change over time [42–44]. Our results showed that beri honey has a large zone of inhibition against XDR *S.* Typhi isolates, followed by neem honey and sidr honey. At the same time, the lowest activity was noted in mustard honey. No information is available to date on the antibacterial activity of Pakistani honey against XDR *S*. Typhi pathogens. However, a previous study conducted in Lahore on the antimicrobial properties of beri honey against MDR *S*. Typhi showed significant antibacterial activity (11–15%) [21]. Recently, a study from Saudi Arabia also reported that different honey samples showed zones of inhibition ranging from 19 to 25 mm against *S*. Typhi isolates. As a topical agent, manuka honey can be used effectively to treat conditions such as atopic dermatitis, blepharitis, rhinosinusitis, and cutaneous ulcers [45]. In a clinical trial in Pakistan, beri honey progressively reduced the bacterial load and effectively healed wounds [46]. In another study, manuka honey showed a zone of inhibition of 7.4 mm against NDM-1-producing Gram-negative blood isolates [47]. We found that beri and neem honeys inhibited and killed some *S.* Typhi isolates at concentrations of 3.125 and 6.25%, respectively. In an Indian study, *Apis indica* honey showed bactericidal activity at a concentration of 3% (v/v) against *S.* Typhi [48]. In a Pakistani study, manuka honey inhibited and killed the NDM-1 producing *Klebsiella pneumoniae* at a concentration of 30% v/v [25]. A recent study from the USA reported an MIC range of 21–27% for manuka honey against the Enterobacterales [49]. Previously published data suggested that cinnamaldehyde, carvacrol, and honey inhibit the expression of *exoS* and *ampC* antibiotic resistance genes in MDR *P. aeruginosa*. Additionally, cinnamaldehyde inhibits pathogenic bacterial growth by disrupting electron transport chains. The beneficial properties of these compounds can make them effective agents for overcoming antibiotic resistance [50]. Additionally, honey contains secondary metabolites such as phenolic compounds, which may contribute to its antibacterial properties. These include syringic acid, which stresses cell membranes; p-coumaric inhibits binding to bacterial DNA; apigenin, chrysin, kaempferol, and galangin, which inhibit the synthesis of bacterial peptidoglycans and ribosomes [51]. The antibacterial activity of honey is influenced mainly by its physiochemical parameters, such as an acidic pH and osmotic pressure, which are the primary factors responsible for its antibacterial properties. However, other factors are also strongly linked to the antibacterial capabilities of honey, such as its hydrogen peroxide content and other non-corrosive components, such as methylglyoxal, the antimicrobial peptide bee defensin-1, polyphenols, and other compounds from bees [52]. In the developing world, honey can be one of the best remedies for skin and stomach pathogens such as *S. aureus*, *E. coli*, and *S*. Typhi. It is readily available at low prices. Our study demonstrates the value of indigenous honeys as antimicrobial agents and the need for further investigations of other types of honey against a variety of antimicrobial-resistant pathogens. Due to time and resource constraints, the study had some limitations, including the lack of ability to detect the stability of each honey, and the inability to determine the contents of local honey by high-performance liquid chromatography (HPLC).

## Conclusion

We report isolates of XDR *S.* Typhi resistant to ampicillin, ciprofloxacin, and ceftriaxone, with high MICs, and sensitive only to azithromycin and carbapenems. Compared to other native honeys, beri (*Ziziphus mauritiana*) honey and neem (*Azadirachta indica*) honey show a potential antibacterial effect, with low MICs and MBCs, against XDR *S*. Typhi isolates carrying *pltB, bla*_CTX-M-15_*, bla*_TEM-1_*, qnrS, qnrA, qnrB,* and *Sul1* genes. As antimicrobial-resistant pathogens are rising, natural remedies for treating various bacterial infections may offer the most promising solutions after in vitro and in vivo clinical trials are conducted to demonstrate their efficacy. The spread of XDR *S.* Typhi pathogens poses a risk due to the possibility that drug-resistance genes may be passed between *S.* Typhi and other strains of bacteria, resulting in highly drug-resistant pathogens. It is possible to prevent the spread of XDR *S.* Typhi by improving hand hygiene and the safety of drinking water and food and vaccination.

## Data Availability

All the data supporting the findings are presented in the manuscript.

## Abbreviations

XDR: Extensively drug-resistant
*S.* Typhi: *Salmonella enterica* Serovar Typhi
MICs: Minimum inhibitory concentrations
MBCs: Minimum bactericidal concentrations
WHO: World Health Organization
MDR: Multidrug-resistant
ARGs: Antimicrobial resistance genes
SSA: *Salmonella Shigella* Agar
MHA: Mueller Hinton agar
AMP: Ampicillin
SXT: Co-trimoxazole
CIP: Ciprofloxacin
CRO: Ceftriaxone
AZM: Azithromycin
IPM: Imipenem
MEM: Meropenem
